# Clinical compatibility of magnetic resonance imaging with magnetic intramedullary nails: a feasibility study

**DOI:** 10.1007/s00402-024-05210-y

**Published:** 2024-02-14

**Authors:** Joseph D. Femino, Samuel R. Barnes, Scott C. Nelson, Lee M. Zuckerman

**Affiliations:** 1 Department of Orthopaedic Surgery, University of Southern California Keck School of Medicine, 1520 San Pablo St., Suite 2000, Los Angeles, CA 90033 USA; 2 Department of Radiology, Loma Linda University Medical Center, Loma Linda, CA USA; 3 Department of Orthopaedic Surgery, Loma Linda University Medical Center, Loma Linda, CA USA

**Keywords:** Precice nail, Precice Bone Transport Nail, Magnetic intramedullary lengthening nails, Magnetic resonance imaging (MRI) compatibility, Distraction osteogenesis, Limb lengthening

## Abstract

**Introduction:**

The use of magnetic resonance imaging (MRI) with a magnetic intramedullary lengthening nail in place is contraindicated per the manufacturer due to the concern of implant activation and migration. A prior in vitro study did not confirm these complications only noting that a 3.0 T MRI weakened the internal magnet. Therefore, a retrospective analysis of patients who underwent an MRI with a magnetic nail in place was performed to determine if any adverse effects occurred in the clinical setting.

**Materials and methods:**

A retrospective review of all patients who underwent an MRI with a magnetic lengthening nail in place was performed. The time spent being imaged in the MRI, number of times the patient entered the MRI suite, and the images obtained were recorded. Radiographs were performed before and after the MRI to determine if any hardware complications occurred. The patients were monitored for any adverse symptoms while they were in the suite.

**Results:**

A total of 12 patients with 13 nails were identified. Two patients underwent imaging with a 3.0 T MRI while the remaining 10 underwent imaging with a 1.5 T MRI. Each patient entered the MRI suite 2.1 times and spent an average of 84.7 min being imaged in the MRI (range 21–494). No patients noted any adverse symptoms related to the nail while in the suite and no hardware complications were identified.

**Conclusion:**

MRI appears to be safe with a magnetic nail in place and did not result in any complications. Given the manufacturer’s recommendations, informed consent should be obtained prior to an MRI being performed and a 3.0 T MRI should be avoided when possible if further activation of the nail is required.

## Introduction

The use of magnetic intramedullary nails for limb lengthening and distraction osteogenesis has significantly increased over the past decade [1–3]. The Precice nail (NuVasive Specialized Orthopedics, Inc., Aliso Viejo, CA, USA) and Precice Bone Transport Nails (BTN) (NuVasive Specialized Orthopedics, Inc., Aliso Viejo, CA, USA) utilize an internal magnet that interacts with an external remote control (ERC) that generates an electromagnetic field to power a motor for bone transport [4, 5]. Both antegrade and retrograde transport are possible based on the orientation of the ERC. The Precice nail is composed of titanium (Ti-6Al-4v) whereas the BTN is made of stainless steel with a BioDur 108 alloy. The nails have been used for multiple indications, including limb lengthening with or without deformity correction, distraction osteogenesis, allograft incorporation, and nonunion repair. The implants have been used in the post-traumatic setting, for congenital syndromes, oncologic indications, and cosmetic purposes.

Due to the internal rare earth magnet, magnetic resonance imaging (MRI) is considered contraindicated while the nail is in place. As the internal magnet is activated using an external magnet in the ERC, there is concern that the magnet in the MRI suite could cause inadvertent activation of the internal magnet with lengthening or shortening of the device, cause pain, migration or displacement of the device by pulling on the magnet, or cause increased heat in the implant from the radiofrequency fields. Current warnings from the manufacturer note the implants are not safe in the MRI suite as this has not been tested in humans [6]. Many patients may need an MRI for clinical indications, and based on current recommendations, the implants would have to be removed prior to these tests being performed. This can be problematic if the patient is actively undergoing treatment with the implant in place or is unable to have the implant removed once it has been placed. Even if it is possible to remove the device, this still exposes the patient to an additional invasive surgery which is not desirable.

Prior testing has been performed on the MAGEC magnetic spinal growth rods (NuVasive Specialized Orthopedics, Inc., Aliso Viejo, CA) [7–11]. Although an MRI can be performed with these implants in place in certain circumstances, the mechanism in these devices is not the same as in the Precice nail or BTN. An in vitro study performed on the Precice nail demonstrated no issues with heating of the implant, migration, or activation of the implants while undergoing an MRI [12]. Given the findings of this prior in vitro study, a retrospective clinical review was performed in vivo to determine if any adverse effects occurred due to an MRI being performed with these implants in place.

## Materials and methods

A retrospective review of all patients between 2015 and 2022 who had a Precice nail or BTN in place and underwent testing with an MRI was performed. Institutional Review Board approval was obtained for this study and the study was performed in accordance with the ethical standards as laid down in the 1964 Declaration of Helsinki and its later amendments. Due to the findings of the prior in vitro study and manufacturer warnings, it was our institutional policy to obtain informed consent prior to performing an MRI in patients who disclosed that the Precice technology was in place [6, 12]. The institutional procedures to allow and approve off-label use of the MRI were established after the magnetic resonance safety officers and radiology physicians reviewed the literature and evaluated the device construction and materials, and determined that the scans posed a risk to the device, but not the patient. A detailed discussion about the manufacturer’s recommendation regarding an MRI and the findings of the prior in vitro study occurred with the patient or caregiver prior to performing the MRI. The orthopedic surgeon requesting the MRI determined whether the risks were greater than the benefit of performing the MRI, and other imaging modalities were performed if the MRI was not felt to be medically necessary. The risks, benefits, and alternatives of performing the MRI were then thoroughly discussed and documented with consent obtained. All patients were monitored for any symptoms at the site of the implant while in the MRI. If the MRI could not be completed, the reasons for this were recorded. The data collected from each patient included the age at the time of placement of implant and time to the first MRI, sex, diagnosis, and indication for placement of the implants, size and location of the implant, and any effects felt by the patient while in the MRI suite.

The time spent while being imaged in the MRI was recorded in addition to the number of times in the MRI suite, strength of the MRI magnet, the part of the body imaged, and sequences performed. Radiographs prior to and after the MRI were also evaluated to determine if there was any activation or migration of the implant. If the patient was still activating the motor after the MRI for transport, they were monitored for any failure of continued transport or other complications.

The time to final follow-up was calculated as the time the implant was placed to the most recent imaging of the implant or implant removal. Final radiographs were also compared to imaging prior to the MRI to determine if any failure of the implant occurred.

## Results

A total of 12 patients were identified with a total of 13 implants placed. The patient’s average age at time of the implant placement was 43 years (range 11–74 years) and first MRI was 45 years (range 12–74 years). The average time from placement of the implant to the first MRI was 23 months (range 3 weeks to 57 months). The average time to follow-up was 40 months (range 4–60 months).

Twelve Precice nails were placed and one BTN. Indications for placement of the implants included after cancer resection in nine patients, lengthening for fibular hemimelia in two patients, and lengthening for proximal femoral focal deficiency in one patient. The implants were used in three patients for limb lengthening using a total of four implants, six patients for compression of an intercalary allograft, in two patients for distraction osteogenesis using plate-assisted bone segment transport (PABST), and in one patient for distraction osteogenesis using a BTN nail.

A total of eight implants were placed on the right side of the patient with five on the left. Nine implants were placed into the femur, one in the tibia, and three in the humerus. Seven of the implants were anterograde trochanteric femoral nails, three were retrograde femoral nails, and three were humeral nails. Three nails were 8.5 mm in diameter, eight were 10.7 mm, one was 11 mm, and one was 12.5 mm.

A total of 38 separate MRI protocols were performed including on the brain, shoulder, elbow, cervical, thoracic, and lumbar spine, chest, abdomen, pelvis, hip, femur, and whole body. Each patient averaged 3.1 separate MRI protocols (range 1–10) including one patient who underwent four whole body MRIs. Multiple sequences were obtained during each protocol including T1, T2, proton density, diffusion weighted, and contrast enhanced imaging. The average number of times each patient entered the MRI suite was 2.1 (range 1–7). The average total amount of time each patient spent in the MRI suite was 84.7 min (range 21–494) whereas each individual session averaged 35 min (range 20–70.6). Two patients underwent imaging with a 3.0 T MRI including the patient with the BTN whereas the remaining were imaged with a 1.5 T MRI. Two separate implants were directly imaged in the field of view of two patients during a total of five sessions. This included the patient who underwent the whole body MRIs on four separate occasions.

Four of the patients were not able to complete at least a portion of their imaging. This included two patients who became claustrophobic and two patients who were unable to tolerate positioning in the MRI due to fractures at sites separate from the extremity with the implant in place. None of the patients noted any pain, warmth, or other symptoms at the site of the magnetic nail while in the MRI suite or during imaging. Three of the patients with incomplete MRIs, including both patients who did not complete an exam due to claustrophobia, were able to successfully complete an MRI at a later time. Another MRI was not attempted in the remaining patient.

**Fig. 1:**
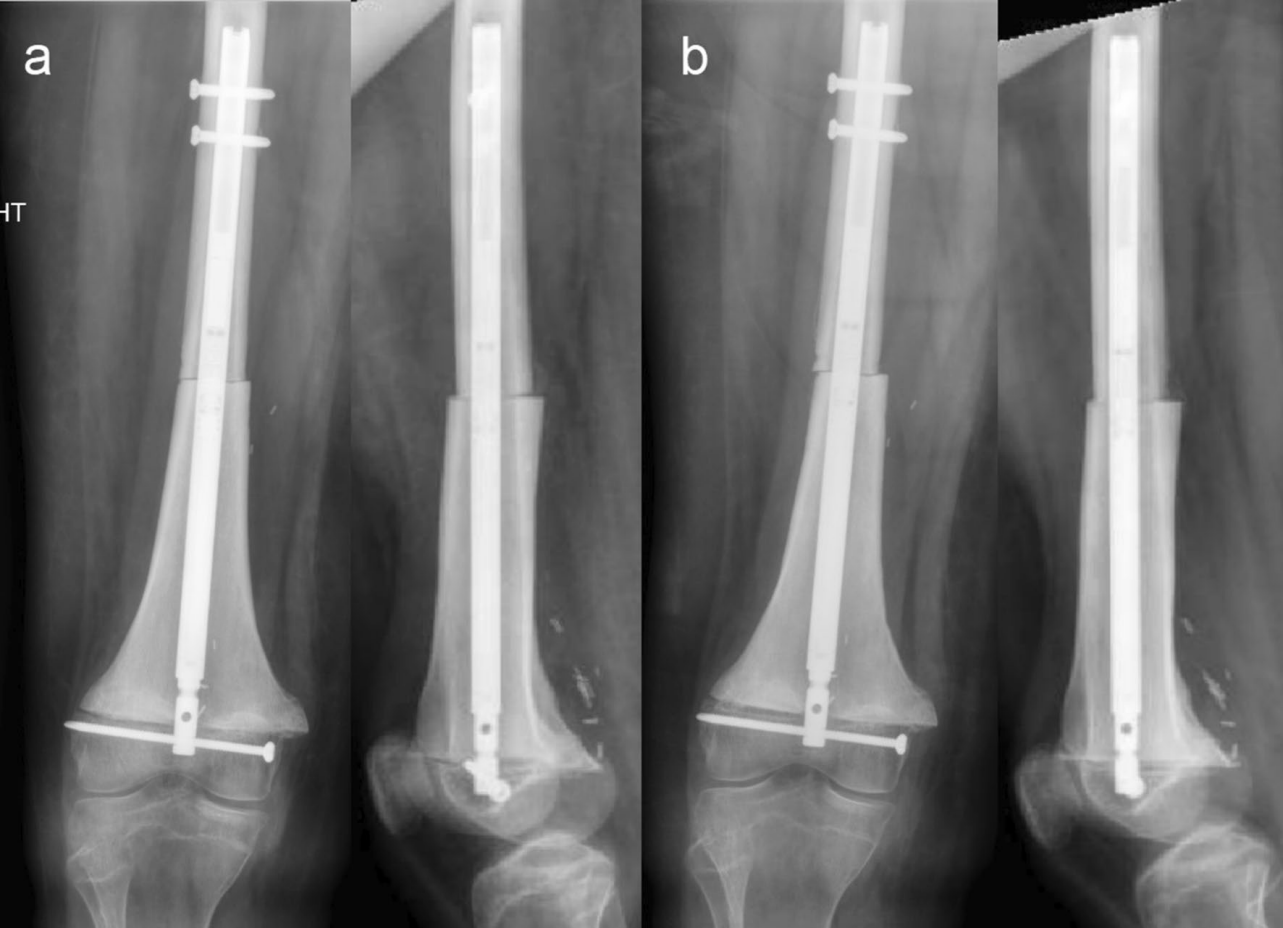
Anteroposterior and lateral radiographs of the distal femur after tumor resection and allograft reconstruction undergoing compression prior to MRI (**a**). Anteroposterior and lateral radiographs immediately after the MRI demonstrate no evidence of activation of the nail or hardware failure (**b**)

**Fig. 2:**
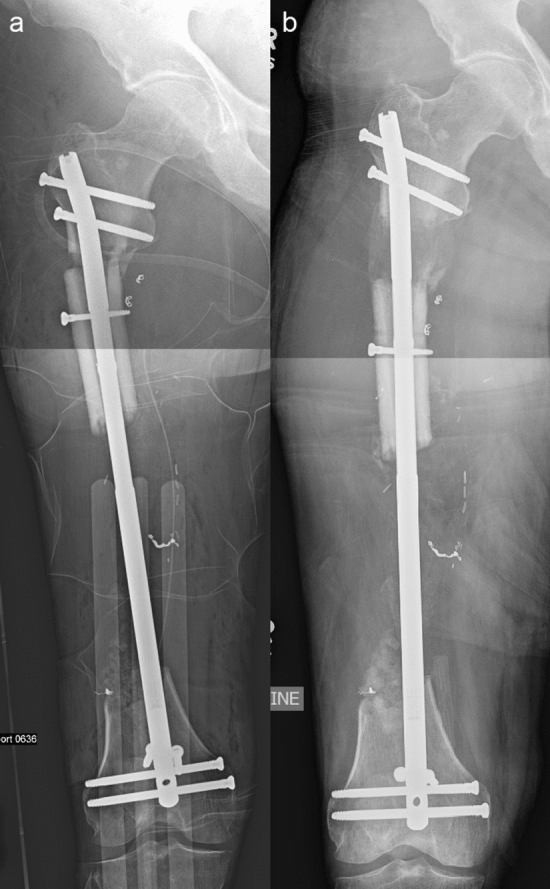
Anteroposterior radiographs of the right femur of a Bone Transport Nail prior to imaging with a 3.0 T MRI (**a**). Forty-five days after the imaging was completed, anteroposterior radiographs demonstrate that the nail was still able to be activated and continue bone transport (**b**)

**Fig. 3:**
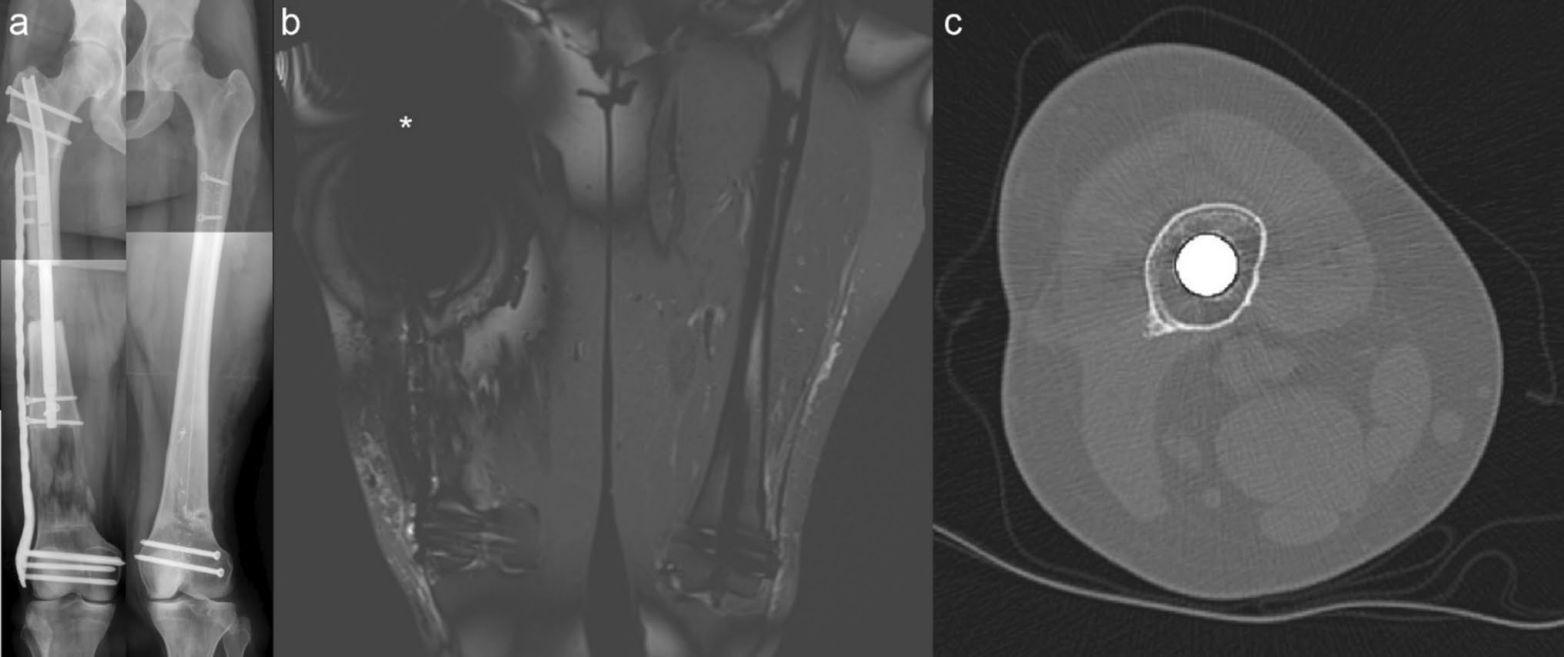
Anteroposterior radiographs of the bilateral femurs demonstrate the right femur undergoing plate-assisted bone segment transport with a titanium plate (Stryker Corp., Kalamazoo MI, USA) and fixation of the left femur with a carbon fiber nail (CarboFix Orthopedics Ltd., Herzeliya, Israel) (**a**). A coronal STIR MRI of the bilateral femurs demonstrates significant artifact surrounding the internal magnet of the Precice nail (*) compared to the plate and carbon fiber nail (**b**). An axial CT of the femur directly through the internal magnet of the Precice nail demonstrates minimal artifact with adequate visualization of the surrounding tissues (**c**)

Radiographs of the implant after the MRI was completed demonstrated no change in all of the patients (Fig. 1). Eight of the patients had not completed treatment with the nail at the time of the MRI and were able to still activate the motor after the MRI was completed. This included two patients who underwent imaging with a 3.0 T MRI (Fig. 2). Due to the internal magnet, if the implant was directly imaged with the MRI, there was a significant artifact created (Fig. 3). This prevented the ability to image the region of the body where the magnet was in place. Otherwise, no adverse effects were identified clinically or at final follow-up on radiographs.

## Discussion

Non-invasive limb lengthening and distraction osteogenesis have the advantages of decreased scarring and less psychological effects compared to traditional external fixation [13–15]. Non-invasive nails that utilized a ratcheting system were difficult to control and could result in inappropriate distraction [16]. Development of the Fitbone (Orthofix Medical Inc., Lewisville, TX), which is driven by a transcutaneous electrical conduit, facilitated significant improvement of the control of the lengthening [17, 18]. The Precice nail subsequently provided the ability to both expand and contract the telescoping portion of the nail which allows for antegrade and retrograde distraction osteogenesis in addition to compression and expansion [19–22]. The Precice nail has been used for distraction osteogenesis using the PABST technique with subsequent development of the BTN [23–25]. The reliability and flexibility of the Precice technology has resulted in a significant increase in non-invasive distraction osteogenesis [16, 26, 27]. Compared to other technology for lengthening, an MRI is either deferred while the implant is in place, or the implant is removed routinely [12].

Based on the prior in vitro study on the Precice nail in the MRI setting, there is no definite contraindication of having the technology in place while in the MRI suite [12]. The authors noted that they did not pre-distract any of the nails, and could not confirm that inadvertent compression of the telescoping mechanism could occur. They also found that a 3.0 T MRI weakened the internal magnet whereas a 1.5 T MRI had no effect. Given these findings, they recommended that if a patient was still undergoing treatment with the nail, then they should avoid a 3.0 T MRI and only have a 1.5 T MRI as this might affect the activation of the magnet in vivo. In our series, two patients underwent a 3.0 T MRI but were still able to continue transport and the MRI did not affect their clinical outcome. Although the rate of transport after the MRI appeared to be appropriate, it is possible that this was not the case as a minor disturbance in the transport rate may not have been measured accurately. None of the nails that were removed in this study were tested to determine if the magnet had any effect on the force that the implant was able to generate. Although there was no adverse effect on the two patients in this study, we would recommend that if a patient is still undergoing transport, a 3.0 T MRI should be obtained with caution and only if medically necessary due to the theoretical risk of damage to the nail. This current study is limited as none of the nails that were removed were evaluated for any decrease in the strength of the implant. Further studies are necessary to determine whether the decrease in strength of the motor occurs in the in vivo setting and whether this is clinically relevant.

Even though none of the patients developed any symptoms localized to the site of the implants while in the MRI suite, imaging of the area of the magnet was unable to be performed due to significant artifact. Due to this, if the patient requires imaging of the region where the magnet is localized, the implant should be removed or other modalities such as a CT scan or ultrasound should be performed (Fig. 3).

Another limitation of this study includes the small sample size and it is possible that some patients may experience symptoms while in the MRI suite. Still, this study has the advantage of evaluating patients of various ages, sizes of nails, and varying MRI protocols. In addition to this, the amount of time each patient spent in the MRI and number of times that the suite was entered helps to verify that an MRI is not contraindicated in these patients. Given this limitation and off-label use of the implant, informed consent should be obtained prior to the patient entering the MRI suite and patients should be closely monitored for any symptoms. Pre- and post-MRI radiographs or other imaging modality should also be obtained to confirm that there were no adverse effects.

Off-label use of an MRI with the Precice technology in place is another concern in terms of liability and ethics. Off-label use of an implant is not considered illegal but should only be performed when the indication is considered reasonable and within the standard of care to minimize both liability to the physician and harm to the patient [28, 29]. The off-label use should be disclosed to the patient and the risks, benefits, and alternatives should be thoroughly discussed. Multiple precedents of off-label use of medications and joint replacement implants in orthopedic surgery as well as the use of MRI with spinal cord stimulators exist [30–35]. Off-label use of implants in the pediatric population is also common as companies may not pursue regulatory approval due to the significant time and costs that are required [36, 37]. To minimize risk and ensure the off-label use is ethical and reasonable, there should be data demonstrating safety in addition to the medical need and benefit it would provide to the patient. In this case, the prior in vitro study and prior studies on the MAGEC rods have demonstrated that the implants are likely to be safe in the MRI setting [7–12]. Scientific reports regarding off-label use can also result in regulatory approval. Based on the prior studies on the MAGEC rods, conditional approval for use of MRI with these implants in place was granted in 2016 [9, 38]. Similarly, multiple clinical studies discussing the use of the Precice nail in pediatric patients were performed prior to regulatory approval, and these nails were only approved for use in the 12-to-18-years age range in the year 2023 [3, 6, 15, 39–41].

The findings of this study help to confirm that an MRI is not a contraindication for patients with the Precice technology in place, although imaging within the region of the internal magnet is not feasible given the significant artifact produced. Further research is required to determine whether other devices that are contraindicated for use with an MRI can be used with the Precice technology. When medically required, imaging with an MRI appears to be safe in these patients. If there are still plans to activate the nail, this should ideally be performed using a 1.5 T MRI and after informed consent is obtained with close monitoring of the patients for any complications.

## Data Availability

The datasets used or analyzed during the current study are available from the corresponding author upon reasonable request.
